# A measure development study of sugar-sweetened beverage-related knowledge, self-efficacy, and intention among urban, low-income adults

**DOI:** 10.1186/s12889-020-10073-0

**Published:** 2021-01-07

**Authors:** Brenda Heaton, Julie A. Wright, Julia C. Bond, Lisa M. Quintiliani

**Affiliations:** 1 Department of Health Policy and Health Services Research, Boston University Henry M. Goldman School of Dental Medicine, 560 Harrison Avenue, 3rd floor, Rm 336, Boston, MA 02118 USA; 2 Department of Epidemiology, Boston University School of Public Health, 560 Harrison Avenue, 3rd floor, Rm 336, Boston, MA 02118 USA; 3 Department of Exercise and Health Sciences, University of Massachusetts Boston, Boston, MA USA; 4 Section of General Internal Medicine, Boston University School of Medicine, Boston Medical Center, Boston, MA USA

**Keywords:** Health status disparities, Surveys and questionnaires, Psychometrics, Adults, Factor analysis, Public housing, Sugar-sweetened beverages

## Abstract

**Background:**

Sugar-sweetened beverage (SSB) consumption is an important behavior that can influence individuals’ risk for diabetes, obesity, and other chronic diseases. Nonetheless, there is a lack of valid measures to assess SSB-related constructs. Reliable and valid measures can help evaluate the efficacy of interventions designed to curb SSB consumption. Our aim was to develop a valid and reliable instrument to measure constructs related to SSB consumption in English and Spanish.

**Methods:**

A cross-sectional survey was conducted among a convenience sample of 150 adult residents of public housing developments in Boston, Massachusetts between July of 2016 and January of 2017. All households from two public housing developments were approached by study staff to solicit participation via door-to-door knocking and posted flyers. We developed questions to measure three SSB-related constructs informed by the Social Cognitive Theory: SSB knowledge, and self-efficacy and intention to act on SSB consumption. The questions were pilot tested in the population, and then administered in-person by bilingual study staff in either English or Spanish. Interviews were conducted most often in the participant’s home and less frequently within a community space. Item normality was assessed with descriptive statistics. Difficulty of knowledge items was assessed with percent correct. Construct validity of self-efficacy items was assessed using confirmatory factor analysis (CFA). A model was considered a good fit if confirmatory factor index (CFI) > 0.95, root mean square error of approximation (RMSEA) < 0.05, and standardized root mean square residual (RMSR) < 0.05. Pearson correlations with consumption scores assessed criterion validity, and intraclass correlation coefficients (ICC) assessed test-retest reliability.

**Results:**

Of the eight knowledge items tested, only four items resulted in correct answers less than half of the time. CFA resulted in a 5-item scale with excellent fit indices (CFI = .99; RMSEA = .025: SRMR = .02) and Cronbach’s (0.79), test-retest (ICC = 0.68), and significant correlation with intention and SSB consumption. Of the four intention items, one was significantly correlated with SSB consumption.

**Conclusions:**

This study provides evidence for the validity of key constructs related to SSB consumption for use in adults, which could provide important theory-based markers for program evaluations of SSB-related interventions.

**Supplementary Information:**

The online version contains supplementary material available at 10.1186/s12889-020-10073-0.

## Background

Sugar-sweetened beverages (SSBs) are a broad category of beverages with added sugars that includes, but is not limited to, soft drinks, fruit juice, energy and sport drinks, and sweetened coffees and teas. They contribute 39% of all added sugars consumed in the United States [1]. High SSB consumption is associated with serious health consequences for children, including weight gain, overweight, obesity, and dental caries [2–4]. Among adults, additional health risks associated with SSB consumption include metabolic syndrome, type 2 diabetes [5], cardiovascular disease [6], and mortality [7].

In light of its deleterious health effects, SSB consumption has been the focus of a diverse array of public health interventions that have been implemented at both the population/community- and individual–levels in an effort to curb population levels of consumption. Population-level interventions have included increasing taxes on SSBs [8] or substituting other beverage options for SSBs in hospitals [9] or schools [10], while efforts to intervene at the individual level have primarily focused on those social and/or cognitive constructs believed to be related to SSB consumption, including knowledge of SSB health effects and attitudes and intentions about SSB consumption [11–14].

While population-level interventions have generally been successful at reducing SSB consumption, individual-level interventions have reported mixed results, likely due to the variation in behavioral choices seen among individuals and across populations, as well as due to differences in how behavioral constructs are measured. Specifically, individual-level interventions have reported an association between increased SSB-related knowledge and decreased consumption [15–17], while others have not [11, 14, 18, 19]. Only one behavioral intervention reported changes in SSB intentions as well as consumption [13]. Importantly, these studies did not use validated scales to measure changes in SSB behavioral constructs. Rather, they used non-validated survey questions related to SSB constructs [15–17, 19] or validated questions focused on SSB-related outcomes such as oral health knowledge and behaviors [11, 14]. The inconsistency of findings underscores the importance of developing valid measures of SSB-related behavioral constructs, used consistently across populations, as an important step in advancing intervention science aimed at reducing population consumption of SSBs.

Systematic reviews and other meta-analyses demonstrate the relationship between theoretical constructs and nutrition-related health behaviors and weight control [20, 21]. There is a lack of standard, valid measures of SSB-related behaviors generally, but particularly among adults. Moreover, there is a lack of measures developed for low-income adult populations despite the fact that these populations bear the greatest burdens of chronic disease and likely need the greatest amount of behavioral support around health-related decision-making [22]. The lack of valid measures may have contributed to the mixed results of individual-level interventions. Further, it has likely precluded a thorough understanding of the behavioral pathways associated with SSB consumption. The development of valid measures of behavioral constructs related to SSB consumption could both help clarify behavioral mechanisms that inform SSB consumption patterns and facilitate assessment of the efficacy of future individual-level interventions targeting SSB consumption.

This study aims to develop valid and reliable questionnaire items and/or scales for three SSB-related constructs informed by the Social Cognitive Theory (SCT). The SCT is a theory encompassing the interactions between environment, people, and their behavior [23, 24]. For these reasons, SCT is frequently used as a theoretical underpinning for SSB-related interventions among both adults [25] and adolescents [12]. In this study, we focused on three constructs most applicable to individual-based interventions, specifically 1) SSB-related knowledge, 2) Self-efficacy to limit SSB intake, and 3) Intention to limit SSB intake. Together, these constructs inform health-related behavior. Knowledge can work in multiple ways, for instance increasing one’s behavioral capability to perform a behavior. Behavioral capability in turn is one factor related to increasing one’s self-efficacy, or ability to perform a behavior. Intention to engage in behavior influences self-regulatory processes such as goal setting and evaluating goal attainment progress.

This paper reports on the development and testing of measures to assess these three theory-related constructs associated with adult SSB consumption.

## Methods

The research plan for this measure development study was reviewed and approved by the Boston University Medical Center Institutional Review Board. Data for the study was collected between July of 2016 and January of 2017. Participants were adult residents of public housing living in two housing developments in Boston, MA.

### Questionnaire development

Questionnaire development was iterative and occurred in multiple phases to facilitate ongoing improvement of the instrument. The research literature was reviewed to identify items that pertained to the behavioral theory constructs of interest – knowledge, self-efficacy, and intention. The research team, which included expertise in nutrition, behavioral science, and epidemiology, reviewed each item to determine the appropriate fit within each construct and ensure that each question was well designed. Once existing items were compiled and reviewed, each construct was assessed to identify whether or not there were potential gaps in measurement. When gaps were identified, such as a lack of SSB-specific knowledge questions or re-framing for an adult population, measures were developed or modified to address them. The methods applied for each construct are described below.

#### Knowledge

Existing questionnaires [12, 15, 26], the majority of which were developed for youth, were reviewed and adapted. New questions were written based upon generally accepted, publicly-available SSB-related knowledge [27–29] and the expertise of the research team. At least one knowledge item corresponding to each self-efficacy and intention question of interest was included. This ensured that the overall questionnaire was asking about knowledge, self-efficacy, and intention related to the same concepts. The initial knowledge instrument had 9 total questions pertaining to adult SSB-related knowledge. Response options were multiple choice, and varied from True or False to more complex responses depending on the question. For all questions, there was only one correct option. A correct response was assigned a 1 and an incorrect or ‘don’t know’ response was assigned a 0.

#### Self-efficacy & intention

Existing questionnaires about SSBs, dental health behavior, and obesity were adapted for SSB-related self-efficacy and intention instruments [12, 30–33]. Questions that had previously been pilot tested in the priority population were prioritized for inclusion [30]. The final questionnaire included 12 items for self-efficacy and 4 for intention. Response options were scored 1 to 5 and ranged from “Not sure at all” to “Extremely sure” for some self-efficacy questions and “Strongly disagree” to “Strongly agree” for other self-efficacy questions, depending on question content. For intention items, the scale was again 1 to 5 and response options ranged from “Do not want to at all” to “Very much want to.” Thus, lower scores were associated with lower self-efficacy and/or intention with respect to SSB consumption.

The 15-question Beverage Intake Questionnaire (BEVQ-15) was administered in addition to questions pertaining to knowledge, self-efficacy, and intention. The BEVQ-15 is a validated measure of adult consumption of sugar-sweetened beverages and was used to assess total weekly calories from beverages and total calories from SSBs [34–36]. However, to our knowledge, the BEVQ-15 has not been validated in populations with demographics similar to our study population i.e. low-income, urban adults. In the BEVQ-15, the predefined categories of SSBs are as follows: Sweetened Juice Drinks, Regular Soft Drinks, Sweet Tea, Coffee with cream and/or sugar, and Energy Drinks. Additional beverage categories that are listed in the BEVQ-15, but are also not classified as SSBs are: water, 100% fruit juice, whole milk, reduced fat milk, low-fat milk, coffee or tea without cream and/or sugar, beer, hard liquor, and wine. This instrument has been updated since the time our research was completed [37].

After finalization of the initial questionnaire, it was translated into Spanish by study staff who are native Spanish speakers and back-translated by native Spanish speakers who were not involved in the translation process. Whenever possible, previously published Spanish translations of existing questions were used. The full questionnaire is available as a supplementary file.

### Pilot testing

The initial questionnaire was pilot tested with members of the priority population: residents of Public Housing Developments (PHDs) in the city of Boston, MA. Residents of PHDs are predominantly comprised of individuals from racial/ethnic minority groups living below the federal poverty line. Owing to contextual issues, including educational access, community resources, and discrimination, residents have been shown to suffer from disproportionately higher rates of chronic disease for which consumption of SSBs is a primary risk factor [22]. For these reasons, pilot testing was aimed at ensuring comprehension of the questionnaire items, and completeness of constructs given cultural and environmental influences. Using a standardized protocol, two bi-lingual study staff administered the first version of the questionnaire to 12 participants, 6 in English and 6 in Spanish. Following the administration of the questionnaire, participants responded to a series of open-ended questions designed to assess the salience of the questionnaire items. In response to pilot testing, we refined, deleted, and merged questions based on feedback received. For example, we modified the language on item 11 of the self-efficacy instrument to state “when it’s on sale at the store” as opposed to the original language of, “when it’s cheap”.

### Sample selection

Participants were recruited from two different PHDs in Boston, MA, one medium-sized (~ 250 households) and one large (~ 400 households) between July of 2016 and January of 2017. The research team obtained a complete listing of households (no resident information) for both PHDs from the Boston Housing Authority, which formed the sampling frame. Given our intent to engage in exploratory factor analysis, our target sample size was 150 [38]. All households were approached at least once to assess eligibility and interest. Study staff who were bilingual in Spanish and English recruited participants via door-to-door knocking and posting flyers throughout the selected developments. Individuals were eligible if they spoke either English or Spanish, were at least 18 years old, and were residents of the selected PHD site. If an eligible individual agreed to participate, staff members attempted to conduct the interview immediately, or schedule for the near future. Recruitment efforts were conducted at various times of the day to maximize the possibility of contacting eligible individuals, including in the evenings and on weekends. In exchange for participation, residents received a $30 gift card to Target stores. Only one adult from each household was enrolled.

### Data collection

Interviews were primarily conducted in the participant’s home or a common area in the PHD. Interviews were conducted by three, bilingual study staff members following a standardized protocol. The questionnaires were administered via a structured one-on-one interview conducted in the participant’s preferred language. On average, the interview took 38 min to complete. Participants did not see the survey items; however, item response options were printed out and made available to participants for viewing upon administering each item. Paper response sheets were used to record responses during the interview. Subsequently, responses were manually entered into an online database. Data from each interview were entered twice by two different study staff members for quality assurance. Discrepancies were reviewed by a third staff member.

In order to test the reliability of participant responses (test-retest), the final 60 participants were given the opportunity to participate in a repeat assessment to occur 13–15 days after the initial assessment with repeated compensation. Thirty participants completed a repeat assessment.

### Statistical analyses

Analyses were conducted using SPSS Statistics (Version 25; Armonk, NY: IBM Corp, 6/17/2018) and IBM SPSS Amos (Version 24, Wexford, PA; Amos Development Corp,5/13/2019). Descriptive statistics to describe the sample and summary scores of questionnaire items were calculated. Construct validity, the extent to which the construct measures what it is thought to measure [38, 39], was assessed by examining hypothesized relations between variables or dimensions for self-efficacy. Criterion validity, whether the construct is related to beverage consumption, was assessed for knowledge, self-efficacy and intention items. Internal consistency reliability and test-retest reliability were assessed for self-efficacy only.

#### Self-efficacy

The construct validity of the self-efficacy scale was evaluated using confirmatory factor analysis (CFA); which was hypothesized to result in a well-defined factor structure.

Prior to running the CFA, means, medians, modes, ranges, skew, and kurtosis were calculated to assess the normality of the 12 self-efficacy questions. Normally distributed data have a skew and kurtosis near 0 and values less than 2.0 are ideal for factor analysis purposes. The team discussed any questions that were not normally distributed to determine if the item should be removed prior to the data analysis. Items with a mean near the middle of the response range, a larger standard deviation, and skew and kurtosis less than one were considered ideal. Items with means, medians, modes that were close to the ends of the response range, skew or kurtosis above two and narrow standard deviations were considered for removal. Response options included as part of the self-efficacy questionnaire allowed for participants to report if they did not drink SSBs or regular soda (see SE2, SE3 in supplementary file). Twenty-seven participants (18%) reported that they did not consume soda, while 11 participants (7%) reported that their consumption did not include other forms of SSBs. Maximum-likelihood estimation and full information maximum likelihood was used to handle missing data [39].

For the CFA, factor loadings below 0.40 were considered for deletion, and model fit and modification indices were examined to optimize the fit. A model was considered to have good fit if confirmatory factor index (CFI) was greater than 0.95, and both root mean square error of approximation (RMSEA) and standardized root mean square residual (SRMR) were less than 0.05 [40]. CFA were completed in IBM Amos (Version 24, Wexford, PA; Amos Development Corp).

Internal consistency (reliability) of the self-efficacy items was assessed with Cronbach’s alpha, a value >.70 was considered good and suggestive of the items measuring the same construct [38]. Test-retest reliability for the self-efficacy scale score was evaluated using intraclass correlation coefficients (ICC).

#### Knowledge

The criterion validity of knowledge was hypothesized to be those who scored correctly on an item would have lower consumption of SSBs and higher intention to engage in healthful beverage consumption behavior. The percent of participants who responded correctly to each question was calculated. Questions that were deemed to be more “challenging” (< 50% correct responses) were then used to compare average BEVQ-15 scores across response categories to assess whether average consumption was significantly different based on SSB-related knowledge.

#### Intention

Criterion validity of intention was assessed by examining the relationship between intention and behavior, and intention and self-efficacy. A negative relationship was hypothesized for behavior and a positive one for self-efficacy.

## Results

### Participants

Table 1 displays the participant characteristics of the sample. A total of 150 PHD residents participated in the survey. Participants predominantly identified as female (79%), Hispanic (62%), unemployed (57%) and had no more than a high school education (59%), which is reflective of the sociodemographic distribution of heads of households in the population (Boston Housing Authority, unpublished data, 2017). It also largely reflects the demographic distributions of public housing residents in other, urban US cities [41]. The majority of our sample (56%) reported daily SSB consumption.

**Table 1:** Baseline demographics of the population of Boston Housing residents (*n* = 150)

Characteristics	n (%)^**a**^
Sex	
Female	119 (79)
Male	31 (21)
Language(s) spoken	
English	60 (40)
Spanish	48 (32)
Both	42 (28)
Race/Ethnicity	
Black non-Hispanic	45 (30)
White non-Hispanic	4 (3)
Hispanic	93 (62)
Other	8 (5)
Education	
Less than High School	39 (26)
High School/GED	49 (33)
More than High School	61 (41)
Missing	1 (1)
Employment Status	
Full-time	30 (20)
Part-time	34 (23)
Unemployed	86 (57)
Self-rated health status	
Excellent	14 (9)
Very good	27 (18)
Good	59 (39)
Fair	39 (26)
Poor	9 (6)
Missing	2 (1)
Frequency of sugary drinks	
Rarely or never	36 (24)
At least once/week, but not every day	29 (19)
Once a day	29 (19)
≥ 2 times/day	56 (37)
Mean age in years (SD)	45.0 (13.9)

*Abbreviations*: *GED* general education diploma, *SD* standard deviation

^a^Percentages may not add to 100% due to rounding

### Measure development

Table 2 displays the percentage of correct responses on each of the 9 knowledge items. Only 4 knowledge questions (K2, K7, K8, K9) had correct responses less than 50% of the time. Correct responses did not vary by language with the exception of K2 for which 50% of Spanish speakers gave correct responses compared to 33% of English speakers (data not shown).

**Table 2:** Descriptive statistics and percent correct for all knowledge questions

Item	Question code	N	Missing	% correct
K1	Water Recs	150	0	92.0
K2	SSB Recs	150	0	40.7
K3	Sugar Types	150	0	82.7
K4	SSB obesity	150	0	98.0
K5	SSB diabetes	150	0	98.7
K6	SSB cavities	150	0	98.7
K7	Natural sugar	150	0	16.0
K8^a^	Added sugar	150	0	27.3
K9	100% Fruit Juice	150	0	30.7

*Abbreviations*: *K* knowledge, *Recs* recommendations, *SSB* sugar-sweetened beverage

^a^See manuscript text and supplementary file for suggested revision

Table 3 displays the descriptive statistics for the self-efficacy and intention questions. All self-efficacy items had a skew and kurtosis below 2 and no single item needed to be transformed*.* Of the 12 self-efficacy items, SE2 (Less Soda) and SE4 (Explain SSB) had a mean near the end of the range and the median and mode at the top of the range (5), with a narrow standard deviation. Of the 4 intention questions, skew was above 2 for one item (INT1, Water Recs) and kurtosis was above 2 for two items (INT1 and INT 4, Explain SSB). The intention question means were generally above the median. The BEVQ-15 scores were not normally distributed, and subsequently they were transformed by top coding to three standard deviations from the mean (top-coded distributions not shown) [42].

**Table 3:** Descriptive statistics and normality indices for self-efficacy and intention questions and BEVQ-15 measures

Item #	Question code	Statistics							
		n	Missing	Mean	Median	Mode	SD	Skew	Kurtosis
**Self-efficacy (SE)**									
SE1	Water Recs	150	0	3.83	4	5	1.28	−0.72	−0.41
SE2	Less Soda	122	28^a^	4.13	5	5	1.11	−0.86	− 0.34
SE3	Less SSBs	139	11^a^	3.65	3	3	1.15	−0.30	−0.50
SE4	Explain SSBs	150	0	4.21	5	5	1.14	−1.16	0.24
SE5	SSB choice	150	0	3.58	4	5	1.49	−0.62	−1.12
SE6	SSB weekdays	146	4	2.54	2	1^b^	1.41	0.45	−1.22
SE7	SSB weekends	146	4	2.51	2	1^b^	1.41	0.48	−1.21
SE8	SSB household	150	0	3.63	4	5	1.36	−0.63	− 0.88
SE9	SSB restaurant	150	0	3.53	4	5	1.39	−0.52	−1.02
SE10	SSB everyone	150	0	3.53	4	5	1.42	−0.51	−1.10
SE11	SSB sale	150	0	3.43	4	5	1.52	−0.34	−1.46
SE12	SSB cheaper	150	0	3.56	4	5	1.51	−0.49	−1.31
**Intention (INT)**									
INT1	Water Recs	150	0	4.63	5	5	0.81	−2.01	3.15
INT2	Less Soda	123	27^a^	4.05	5	5	1.37	−1.15	0.02
INT3	Less SSB	139	11^a^	3.68	3	5	1.35	−0.54	−0.76
INT4	Explain SSB	150	0	4.41	5	5	1.12	−1.85	2.49
**BEVQ-15**									
–	Total Beverage kcal	150	0	411.77	239.63	0	445.94	2.00	4.62
–	SSB Beverage kcal	127	23^a^	239.83	124.74	65.6	315.3	3.11	13.94

*Abbreviations*: *BEVQ-15* 15-question beverage intake questionnaire, *Recs* recommendations, *SD* standard deviation, *SSB* sugar-sweetened beverage

^a^Indicates number of respondents who reported consuming no SSBs and/or regular soda

^b^Multiple modes present. The lowest is shown

The 12 self-efficacy items had good reliability (α = .85) (*n* = 146). However, 3 pairs of self-efficacy items were deemed conceptually similar and had high inter-item correlations: SE8 (SSB household) and SE10 (SSB everyone) (*r* = .80, *r*^*2*^ = .64), SE6 (SSB weekday) and SE7 (SSB weekend) (*r* = .62, *r*^*2*^ = .38), and SE11 (SSB sale) and SE12 (SSB cheaper) (*r* = .81, *r*^*2*^ = 0.66). The research team agreed to select 1 item from each pair, based on how well it was perceived to reflect the construct’s domain, which reduced the 12 initial items to 9 items.

**Fig. 1:**
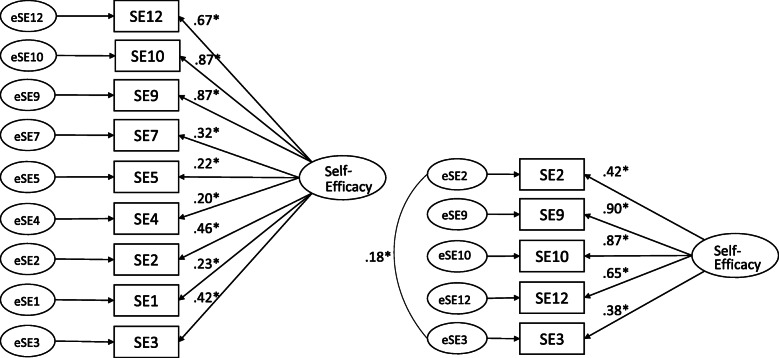
CFA results for the 9- and 5-item models with factor loadings (standardized estimates). Abbreviations: CFA, confirmatory factor analysis; SE, self-efficacy

Confirmatory factor analysis revealed that the 9-item self-efficacy model had poor model fit (χ^2^_[27]_ = 96.93, *P* < .001; CFI = .80; RMSEA = .13 (95% confidence intervals [CI] .10, .16); SRMR = .10.; and, the *p*-value of close fit (pclose), which tests whether the RMSEA is > 0.05, was < .001. Four of the self-efficacy items had factor loadings <.40 (see Fig. 1) and were not significant except SE7 (SSB weekend). A 5-item self-efficacy model that removed the 4 low-loading items model resulted in a better fit as indicated by a significant change in the Chi Square and good fit indices: χ^2^_[4]_ = 4.37, *p* = .36; CFI = .99; RMSEA = .025 (95% CI .00, .13), pclose = .54; SRMR = .02. Factor loadings for the items ranged from .38–.90 (Fig. 1). The results indicate a close model fit. Additionally, the 5-item version had a lower Akaike Information Criterion (26.37) compared to the 9-item version (132.93). All factor loadings were significant, although item SE3 had a factor loading < 0.4. The ICC from the test-retest analysis was 0.68 (95% CI .42, .83) for the 5-item SE measure, suggesting a moderate degree of test-retest reliability.

Results related to criterion validity are presented in Tables 4, 5. Correlations among the 5-item self-efficacy summary score, BEVQ-15 scores, and the 4 intention items are presented in Table 4. There was a significant negative correlation between self-efficacy summary score and total beverage caloric intake and SSB-specific calories. Intention items were negatively correlated with beverage consumption, aside from INT1 (Water Recs), which was positively correlated with SSB consumption (0.014) and INT3 (Less SSB) which was positively correlated with total beverage calories. However, the only significant correlations between intention items and consumption was between INT2 (Less Soda) and total beverage calories (− 0.19), and INT3 (less SSB) and SSB kcal (− 0.18). All intention measures were positively and significantly correlated with the 5-item self-efficacy summary score, aside from INT4 (Explain SSB) which did not reach statistical significance.

**Table 4:** Correlation coefficients between total beverage and SSB-related energy intake (BEVQ-15), Self-Efficacy 5-item summary score, and Intention items

	SE summary score (5 items)	INT1	INT2	INT3	INT4
		(Water Recs)	(Less Soda)	(Less SSB)	(Explain SSB)
**Total Beverage kcal**	−0.4	−0.01	−0.19	0.06	−0.05
n	150	150	123	139	150
*p-value*	*0.001*	*0.89*	*0.04*	*0.49*	*0.52*
**SSB Beverage kcal**	−0.27	0.01	−0.12	−0.18	− 0.09
n	127	127	103	119	127
*p-value*	*0.003*	*0.88*	*0.23*	*0.046*	*0.34*
**SE score**	–	0.18	0.26	0.27	0.05
n	–	150	123	139	150
*p-value*	*–*	*0.03*	*0.004*	*0.001*	*0.51*

*Abbreviations*: *BEVQ-15* 15-question Beverage Intake Questionnaire, *INT* Intention, *Recs* recommendations, *SE* self-efficacy, *SSB* sugar-sweetened beverage

**Table 5:** Mean total beverage and SSB-related energy intake (BEVQ-15) and self-efficacy summary scores by Knowledge item responses

	BEVQ-15		
	Total kcal (*n* = 150)	SSB kcal (*n* = 127)	SE summary score (*n* = 150)
**K2 (SSB Recs)**			
Correct	266.1	139.9	20.47
Incorrect	496.5	285.0	16.97
*p-value*	*0.001*	*0.001*	*0.001*
**K7 (Natural Sugar)**			
Correct	400.6	219.1	17.57
Incorrect	403.2	234.0	18.55
*p-value*	*0.98*	*0.81*	*0.36*
**K8**^**a**^ **(Added Sugar)**			
Correct	440.1	269.9	19.44
Incorrect	388.8	217.8	18
*p-value*	*0.5*	*0.35*	*0.1*
**K9 (100% Fruit Juice)**			
Correct	413.5	260.9	19.13
Incorrect	398.1	218.2	18.07
*p-value*	*0.83*	*0.42*	*0.17*

*Abbreviations*: *BEVQ-15* 15-question Beverage Intake Questionnaire, *K* Knowledge, *Recs* recommendations, *SE* self-efficacy, *SSB* sugar-sweetened beverage

^a^See manuscript text and supplementary file for suggested revision

Table 5 displays mean beverage consumption and self-efficacy summary score for those responding correctly and incorrectly to knowledge items K2 (SSB Recs), K7 (Natural Sugar), K8 (Added sugar), and K9 (100% Fruit Juice). Average consumption of calories from beverages and SSB beverages, specifically, as well as mean self-efficacy summary score differed significantly by responses to knowledge item K2 but not items K7-K9.

## Discussion

This study describes the development and initial validation of a questionnaire to assess 3 constructs related to adult SSB consumption: SSB-related knowledge (4 final items), self-efficacy (5 final items), and intention (4 final items). The results provide evidence for the validity of some of the items. The self-efficacy scale drew upon the literature to develop a novel scale for self-efficacy of sugar-sweetened beverage consumption in adults. The resulting 5-item scale met the criteria used to establish a good fitting model, suggesting good construct validity. Additionally, as predicted, the 5-item self-efficacy summary score was negatively correlated with calories consumed via beverages overall, and SSBs specifically, suggesting criterion validity of the scale, a subset of construct validity. The scale also had good internal consistency (alpha) and moderate test-retest reliability.

While there is extensive literature on self-efficacy [12, 32, 33] the literature is less extensive for knowledge and intention questionnaires. In fact, for knowledge, we were unsuccessful in identifying existing questionnaires to inform this study. Four of the 8 items evaluated were discarded because they were not difficult enough (about 92–99% answered correctly) to provide an evaluation sensitive enough to show gains in knowledge across time. It is possible that these questions were answered correctly because of ongoing campaigns in urban areas in regard to SSB consumption. It is also possible that the use of the True/False format increased the chances of answering correctly. The four remaining items appeared to be more difficult, with 16 to 40% of the sample answering correctly. These items also included 3 true/false questions, but it is possible the content was not as well-known in this population. The knowledge item with the best profile (K2) used a multiple-choice format and 40% answered it correctly. This item was also significantly correlated with the self-efficacy summary score, and in the predicted direction, suggesting good criterion validity, whereas the other 3 items did not show statistically significant correlations. Future research could explore additional knowledge questions to improve the clarity of the wording, especially K8, and increase the breadth with which the construct can be measured. We recommend including all knowledge questions to enable a thorough assessment of the knowledge of different populations.

As mentioned previously, there is scant literature on intention questionnaires for SSB-related behaviors in adults. The criterion validity of the intention items was evaluated in this study by predicting that intention to drink fewer SSB-related beverages, more water, or tell others about what sugary drinks are would be negatively related to actual consumption. The intention to drink less soda (INT2) was significantly correlated with total caloric intake suggesting good criterion validity. It is possible that we did not find additional correlations between intention and consumption because of the “intention-behavior gap”, the idea that reported intentions do not always translate to behavior change [43]. However, there is evidence of convergent validity for all 4 intention items because they were significantly correlated with self-efficacy scores in the expected direction. Taken together the intention items have good evidence for construct validity, although future studies should continue to explore their validity.

This study is the first to design and assess the validity of constructs related to consumption of SSBs in adults. Other studies focused on SSB-related behaviors have often used measures that were not validated, including questions related to SSB knowledge [15, 26], intention [44], and self-efficacy [17]. These constructs are either directly specified or contribute to constructs within the SCT, which is a broad theory encompassing the interactions between environment, people, and their behavior [23]. However, these constructs do not cover all aspects of SCT, which also include important factors such as outcome expectations and observational learning, which could be included in future questionnaire iterations.

Our study has several strengths. Measures were developed, pilot tested, adapted, and evaluated in both English and Spanish in a racially-diverse population of people living in public housing. Public housing residents are more likely than other populations to be from racial and ethnic minority groups and immigrants, and experience chronic diseases and poor overall health [22]. As such, health-related research and interventions that incorporate public housing residents have a high potential to improve important health disparities. Study participants had a wide variety of SSB consumption patterns, with 27 people reporting that they consumed no soda and 11 people reporting no consumption of other SSBs types. There were also a fair number of respondents with high levels of SSB consumption. While the constructs measured by this scale are applicable to broad populations, a strength of this study is its testing in a diverse sample with varied SSB consumption patterns. That being said, future research on these measures would benefit from evaluating potential differences among individuals in racial/ethnic minority groups in SSB and related constructs.

This study should be interpreted in the context of some important limitations. This study was limited by the relatively small sample size of 150 PHD residents. The sample was also primarily women; thus, the validity of these measures may be different among men. The knowledge instrument was also limited by the high proportion of correct answers. Compared to other populations, our sample appeared to have greater knowledge of our assessed constructs [45, 46]. When considering using this scale in other populations, researchers may want to include all of the knowledge questions. Specifically, item K8 had limitations as worded. The question was intended to assess knowledge about the impact of sugars contained in different beverages on dental health. However, as written, it could be read as ambiguous. This is because sugars added to SSBs are molecularly distinct from fructose found in fruit and lactose found in milk, despite the fact that they contribute similarly to dental carries and sugar-related health issues. We would recommend adjustments to this question for future uses of the scale, for example “The sugar that is found naturally in milk can cause cavities, just like sugar that is added to foods and drinks when they are being prepared.” Though this scale is designed to be broadly applicable to adult populations, this measure would benefit from being validated in other populations. Lastly, like any questionnaire study, the results may suffer from some amount of measurement error owing perhaps to one’s ability to recall information or perceptions of what responses may be socially desirable.

## Conclusions

This study provides evidence for the validity of SSB-related knowledge, self-efficacy and intention questions for use in adults, which could provide important theory-based markers for program evaluations of SSB-related interventions. The existence of validated questionnaires is a necessary pre-requisite to understanding the mechanisms by which interventions are working (or not working), allowing program planners to make necessary adjustments for future interventions. These measures could be used in other settings that serve diverse and/or underserved populations similar to public housing developments, such as Special Supplemental Nutrition Program for Women, Infants, and Children (WIC) Centers, safety-net hospitals, and nutrition assistance/food distribution programs. Nevertheless, the validity of these questionnaires should be evaluated in these settings and other populations (e.g., men) to ensure they are generalizable.

## Supplementary Information

**Additional file 1.** Sugar- Sweetened Beverage Questionnaire. Description of data: Table containing the full questionnaire administered to study participants.

## Data Availability

The datasets used and/or analyzed during the current study are available from the corresponding author on reasonable request.

## Abbreviations

BEVQ-15: 15-question Beverage Intake Questionnaire
CFA: Confirmatory factor analyses
CFI: Confirmatory factor index
ICC: Intraclass correlation coefficients
PHDs: Public Housing Developments
RMSEA: Root mean square error of approximation
SCT: Social Cognitive Theory
SRMR: Standardized root mean square residual
SSB: Sugar-sweetened beverage
WIC: Special Supplemental Nutrition Program for Women, Infants, and Children
