# Supplementary material for: A measure development study of sugar-sweetened beverage-related knowledge, self-efficacy, and intention among urban, low-income adults
Source: BMC Public Health. 2021 Jan 7;21:69. doi: 10.1186/s12889-020-10073-0 (PMC7792019; doi:10.1186/s12889-020-10073-0)
Supplement: Supplementary file 1 — Additional file 1. Sugar- Sweetened Beverage Questionnaire. Description of data: Table containing the full questionnaire administered to study participants. [file 12889_2020_10073_MOESM1_ESM.docx]

**Additional file 1**

**Sugar- Sweetened Beverage Questionnaire**

| **Item #** | **Question Code** | **Question and response options** |
| --- | --- | --- |
| **Knowledge (K)** | | |
| K1 | Water Recs | What is the recommended amount of water adults should drink each day?   None   1-2 cups   3-4 cups   5-8 cups   Don’t know   Refused |
| K2^a^ | SSB Recs | What is the recommended limit on the number of sugary drinks (for example: regular soda, energy drinks) adults should drink each day?   0 sugary drinks   1 sugary drink   2 sugary drinks   3 sugary drinks   Don’t know   Refused |
| K3 | Sugar Types | Which of the following are types of sugar?   Corn syrup   Honey   Fructose   Molasses   These are all sugars   Don’t know   Refused |
| K4 | SSB obesity | Frequent consumption of sugary drinks increases the risk of obesity   True   False   Don’t know   Refused |
| K5 | SSB diabetes | Frequent consumption of sugary drinks increases the risk of diabetes   True   False   Don’t know   Refused |
| K6 | SSB cavities | Frequent consumption of sugary drinks increases the risk of cavities   True   False   Don’t know   Refused |
| K7^a^ | Natural sugar | Natural sugar, like honey, is healthier for teeth than white sugar.   True   False   Don’t know   Refused |
| K8^a,b^ | Added sugar | The sugar that is found naturally in fruits and milk is the same as sugar that is added to foods and drinks when they are being prepared.   True   False   Don’t know   Refused |
| K9^a^ | 100% Fruit Juice | Sugar that is found in 100% fruit juice is healthier than the sugar that is added to make soda.   True   False   Don’t know   Refused |
| **Self-Efficacy (SE)** | | |
| SE1 | Water Recs | How sure are you that you can drink the recommended 8 cups of water each day?  [1] Not sure at all  [2]  [3] Somewhat sure  [4]  [5] Extremely sure   Don’t know   Refused |
| SE2^a^ | Less Soda | How sure are you that you can drink less regular soda?  [1] Not sure at all  [2]  [3] Somewhat sure  [4]  [5] Extremely sure   I do not drink regular soda   Don’t know   Refused |
| SE3^a^ | Less SSBs | How sure are you that you can drink less of other types of sugary drinks that aren’t soda (fruit juice, iced tea, lemonade, sports drinks, energy drinks, sugar-sweetened tea or coffee)?  [1] Not sure at all  [2]  [3] Somewhat sure  [4]  [5] Extremely sure   I do not drink sugary drinks   Don’t know   Refused |
| SE4 | Explain SSBs | How sure are you that you can explain to your family and friends what sugary drinks are?  [1] Not sure at all  [2]  [3] Somewhat sure  [4]  [5] Extremely sure   Don’t know   Refused |
| SE5 | SSB choice | When I have a choice it is easy for me to choose diet or unsweetened beverages instead of sweetened beverages.  [1] Strongly disagree  [2] Disagree  [3] Neither agree/disagree  [4] Agree  [5] Strongly agree   Don’t know   Refused |
| SE6 | SSB weekdays | It would be difficult for me to limit my intake of sugar-sweetened beverages on weekdays.  [1] Strongly disagree  [2] Disagree  [3] Neither agree/disagree  [4] Agree  [5] Strongly agree   Don’t know   Refused |
| SE7 | SSB weekends | It would be difficult for me to limit my intake of sugar-sweetened beverages on weekends.  [1] Strongly disagree  [2] Disagree  [3] Neither agree/disagree  [4] Agree  [5] Strongly agree   Don’t know   Refused |
| SE8 | SSB household | How sure are you that you could keep yourself from having sugary drinks (such as regular soda, energy drinks, sweet tea) when other members of your house drink it?  [1] Not sure  [2] A little sure  [3] Sure  [4] Very sure  [5] Extremely sure   Don’t know   Refused |
| SE9^a^ | SSB restaurant | How sure are you that you could keep yourself from having sugary drinks (such as regular soda, energy drinks, sweet tea) when you eat at a restaurant?  [1] Not sure  [2] A little sure  [3] Sure  [4] Very sure  [5] Extremely sure   Don’t know   Refused |
| SE10^a^ | SSB everyone | How sure are you that you could keep yourself from having sugary drinks (such as regular soda, energy drinks, sweet tea) when it seems like everyone around you is drinking it?  [1] Not sure  [2] A little sure  [3] Sure  [4] Very sure  [5] Extremely sure   Don’t know   Refused |
| SE11 | SSB sale | How sure are you that you could keep yourself from having sugary drinks (such as regular soda, energy drinks, sweet tea) when it’s on sale at the store?  [1] Not sure  [2] A little sure  [3] Sure  [4] Very sure  [5] Extremely sure   Don’t know   Refused |
| SE12^a^ | SSB cheaper | How sure are you that you could keep yourself from having sugary drinks (such as regular soda, energy drinks, sweet tea) when it’s cheaper than bottled water?  [1] Not sure  [2] A little sure  [3] Sure  [4] Very sure  [5] Extremely sure   Don’t know   Refused |
| **Intention (INT)** | | |
| INT1^a^ | Water Recs | How much to you want to drink the recommended 8 cups of water each day?  [1] Do not want to at all  [2]  [3] Somewhat want to  [4]  [5] Very much want to   Don’t know   Refused |
| INT2^a^ | Less Soda | How much to you want to drink less regular soda?  [1] Do not want to at all  [2]  [3] Somewhat want to  [4]  [5] Very much want to   Don’t know   Refused |
| INT3^a^ | Less SSB | How much to you want to drink less of other types of sugary drinks that aren’t soda (fruit juice, iced tea, lemonade, sports drinks, energy drinks, sugar-sweetened tea or coffee)?  [1] Do not want to at all  [2]  [3] Somewhat want to  [4]  [5] Very much want to   Don’t know   Refused |
| INT4^a^ | Explain SSB | How much to you want to explain to your family and friends what sugary drinks are?  [1] Do not want to at all  [2]  [3] Somewhat want to  [4]  [5] Very much want to   Don’t know   Refused |

^a^These items were included in the final measure.

^b^Please consider revising this question for future use to resolve ambiguity. We recommend changing to “The sugar that is found naturally in fruits and milk has the same impact on tooth health as sugar that is added to foods and drinks when they are being prepared.”
